# An Unfortunate Case of Takotsubo Cardiomyopathy During Plasmapheresis for Myasthenia Crisis

**DOI:** 10.7759/cureus.20865

**Published:** 2022-01-01

**Authors:** Abdullah Jahangir, Muhammad Rafay Khan Niazi, Syeda Sahra, Aneeqa Javed, Michael Krzyzak

**Affiliations:** 1 Internal Medicine, Northwell Health, Staten Island, USA; 2 Medicine, Northwell Health, Staten Island, USA

**Keywords:** myasthenia gravis (mg), therapeutic plasmapheresis, plasmapheresis, myasthenic exacerbation, tako-tsubo cardiomyopathy (ttc)

## Abstract

A literature review shows scarce reports of myasthenic crises (MC) complicated by Takotsubo cardiomyopathy (TC). This patient cohort (0.11%) has higher all-cause mortality and prolonged in-hospital course. We present a rare case of a 72-year-old man who developed cardiogenic shock post-plasmapheresis for myasthenia crisis. He became hemodynamically unstable and developed acute respiratory failure requiring intubation 30 minutes after completion of plasma exchange. Serum troponin peaked at 3.19 ng/mL while an emergent 12-lead electrocardiogram (EKG) showed new-onset diffuse ST-segment elevation. Hypokinesis of the entire apex, anterior septum, mid-and apical inferior septum, and mid-and apical inferior wall consistent with Takotsubo cardiomyopathy was seen on bedside echocardiogram. The patient received a continuous infusion of norepinephrine and vasopressin. The hospital course was complicated by multiorgan failure and eventual demise. This case highlights MC and the potential of plasma exchange therapy to induce TC.

## Introduction

Tokutsobu cardiomyopathy (TC) and myasthenia crisis (MC) are two entirely different crises, yet reported many times together. TC is the reversible stunning of the myocardium, which is secondary to the coronary vessels' vasospasm, owing to excessive catecholamine release in the body [1-2]. This surge in the catecholamine release can result from emotional or physical stress to the body that can be in the form of bad news or severe sickness.

Myasthenia gravis is an acquired autoimmune disorder secondary to autoimmune antibodies against nicotinic post-synaptic acetylcholine receptors leading to defective transmission of trans-synaptic action potential causing muscle weakness [2]. Some of the most common precipitating factors of MC are severe illness (e.g., pneumonia), medications (azithromycin by increased circulating catecholamine levels), and emotional and physical stress [3]. Although it is a multisystem disorder, cardiac involvement is seen in 16% of cases. Pericarditis, myocarditis, arrhythmia, and TC are worth mentioning myasthenia gravis-related cardiac complications [4-10]. Previous analysis shows a 15 times greater prevalence of secondary TC following MC than the general inpatient population and twice the odds of all-cause mortality in these patients [11].

Coronary vascular dysfunction can also contribute to the pathogenesis of TC. Sympathetic surge and resultant catecholamine induce vascular constriction and microvascular endothelial cell apoptosis in the affected coronary vessels. Adenosine use causes improvement in the left ventricular function, showing the transient nature of cardiac dysfunction in TC [12-13]. The combined stress of MC and TC can have fatal consequences for the patient. This case stresses the need for repeated electrocardiograms (EKGs), echocardiography, and serial cardiac enzyme measurement in all myasthenia crisis patients.

## Case presentation

A 72-year-old male with a new diagnosis of myasthenia gravis one month ago (compliant with his pyridostigmine regimen 60 mg every 4 hours) was referred to the hospital to evaluate worsening dysphagia. He reported a choking sensation upon attempting to swallow solid foods, difficulty chewing, and a loss of appetite with a consequent 40 lbs weight loss. He also experienced increasing blurry vision towards the end of the day with a noticeably decreased voice volume. He denied any diplopia, weakness, difficulty with ambulation, or gait disturbance.

Physical examination on admission was significant for nasal twang in voice, generalized symmetric facial weakness with reduced cheek puff, weak smile, and difficulty with left eye closure. The patient was vitally stable with a body mass index (BMI) of 21.4 kg/m^2^. He was saturating 96% on room air with no significant findings on routine blood work. On admission, vital capacity (VC) was 550 cc, and negative inspiratory force (NIF) was -35 cm H­­_2_O­ with good effort. The patient deteriorated acutely within a few hours, with VC dropping down to 200 cc with low effort accompanied by worsening muscle weakness. A myasthenic crisis was suspected. He was admitted to the intensive care unit (hospital day 1) for the initiation of plasmapheresis. Plasmapheresis's first dose was administered, 30 minutes after which he became hypotensive (mean arterial pressure < 65 mmHg) and tachypneic. The patient was intubated and started on mechanical ventilation due to acute hypoxemic respiratory failure with hypercapnia. Vasopressor support was initiated due to persistent hypotension.

**Figure 1:**
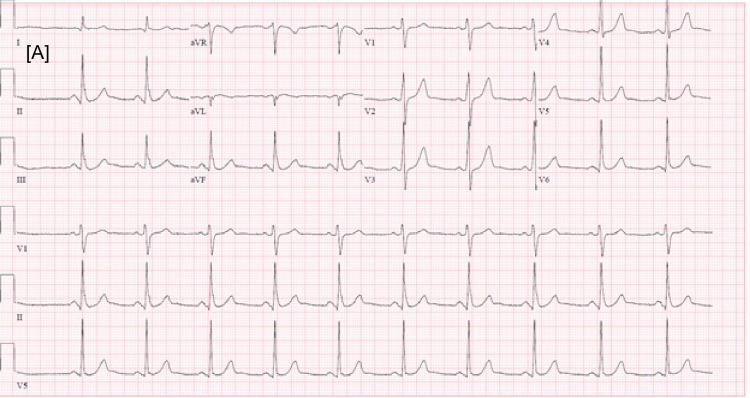
(a) Routine electrocardiogram (EKG) on arrival showing sinus rhythm, PR interval 138 ms, HR 64 beats per minute

**Figure 2:**
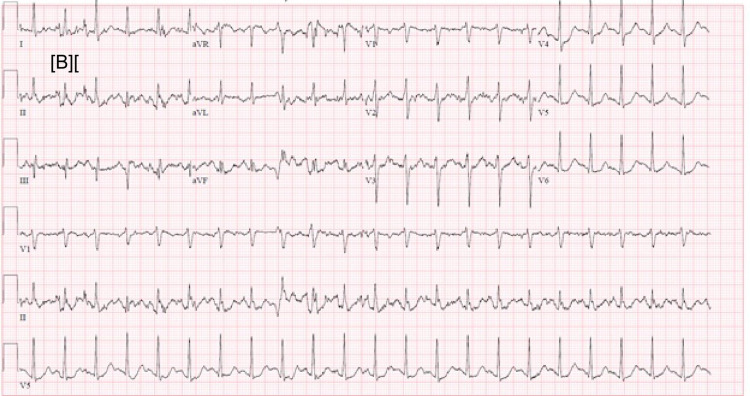
Sinus tachycardia, non-specific ST abnormality, HR: 134 beats per minute, PR interval: 136 ms (1 h after starting plasmapheresis)

**Figure 3:**
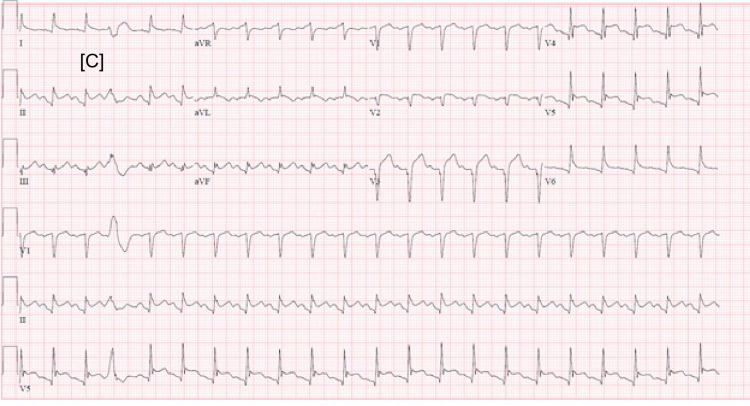
Sinus tachycardia with occasional premature ventricular contractions (PVCs), HR 130, PR 146 ms (4 hours after initiation of plasmapheresis)

Diffuse ST elevations were seen on EKG (Figure 1 shows the EKG on arrival, Figures 2-3 show EKG after starting plasmapheresis).

**Figure 4:**
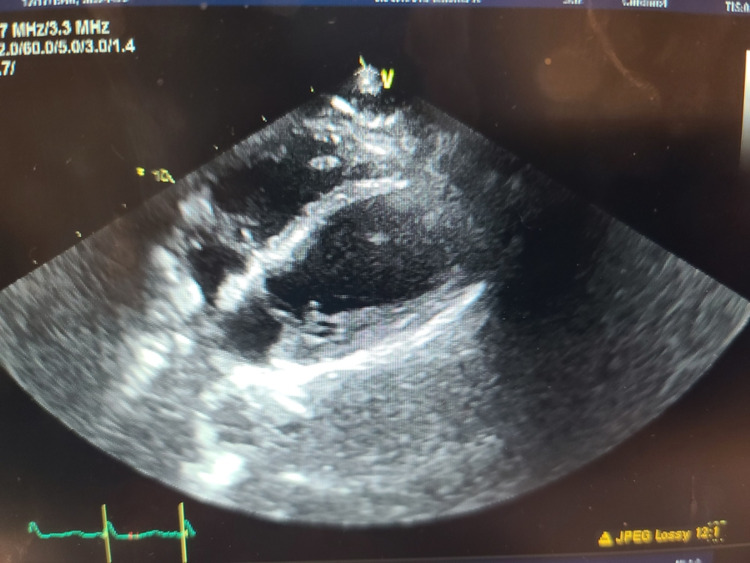
Echocardiogram findings consistent with low ejection fraction and Takotsubo cardiomyopathy

Troponins were elevated at 0.2 ng/mL and continued to rise to 3.19 ng/mL within 24 hours. An echocardiogram showed an ejection fraction (EF) of 12%, severely decreased global left ventricular (LV) systolic function, global cardiomyopathy, moderately reduced right ventricular dysfunction, and an apical thrombus. LV wall motion abnormalities, including hypokinesis of the entire apex, anterior septum, mid and apical inferior septum, and mid and apical inferior wall consistent with Takotsubo cardiomyopathy, were seen (Figure 4, Video 1).

**Video 1:** Echocardiogram findings consistent with a low ejection fraction

Plasma exchange was held due to decompensation. Mestinon and prednisone were continued without any clinical improvement. The clinical status continued to worsen over the next week with multiorgan failure, including persistent hypotension, necessitating continued pressor support (low dose norepinephrine and vasopressin), hypoxic respiratory failure with high anion gap metabolic acidosis (HAGMA) (serum lactate 6.1 mmol/L), new-onset atrial fibrillation, renal failure needing continuous venovenous hemodialysis (CVVH), and hemodialysis (HD). He eventually developed disseminated intravascular coagulation (DIC). NIF continued to fall from -20 to -8. The patient was started on Amiodarone for new-onset atrial fibrillation and a 20-second episode of ventricular tachycardia but, later that day, the patient became bradycardic post HD session and expired after having a cardiac arrest on hospital day 15.

## Discussion

According to a review, the myasthenic crisis begins with generalized weakness, bulbar symptoms, and weakness of respiratory muscles in 76%, 19%, and 5% cases, respectively, according to a review [14]. If we compare the symptoms of the patients with TC and MC looking at previous systemic reviews, most of them proceed to respiratory failure leading to intubation [15].

Elevated plasma catecholamine levels demonstrate cardiac sympathetic activity in TC as compared to acute myocardial infarction patients. A high level of catecholamine in the blood triggers the transformation of signaling in the ventricular myocytes from Gs (stimulatory) to Gi (inhibitory) proteins via beta-2-adrenergic receptors, which has a negative inotropic effect and thus contributes to Takutsubo or stress cardiomyopathy. TC and MC share similar precipitants like emotional or physical stress, infection, and pain, among others [16]. Therefore, a common precipitant could have triggered them together. A systematic review of TC occurring with MC showed that all patients studied experienced myasthenia crisis, hypothesizing that it could be a possible trigger for TC [17].

TC is further divided into four different morphologic types, including the most classic apical type: apical hypokinesis with basal hyperkinesis, basal or inverted type with mid-ventricular or basal hypokinesis with apical hyperkinesis, focal type featuring hypokinesis of a focal myocardial segment, and mid-ventricular type with basal hypokinesis with mid-ventricular hypokinesis with apical hyperkinesis.

The myocardium's unequal contraction in the catecholamine surge setting is attributed to the unequal distribution of beta-adrenergic receptors (BAR) throughout the myocardium. There is decreased density of sympathetic nerve endings at the heart's apex compared to the base. Contrarily, BARs are more concentrated at the heart's apex compared to the base of the heart. This disproportionate distribution of BAR and sympathetic nerve endings throughout the myocardium results in an increased concentration of catecholamine localization at the heart apex. A consequently increased influx of calcium inside myocytes leads to damage of the sarcolemma membrane and affects the contractility of the heart [18].

Our patient was just diagnosed with myasthenia gravis (MG) two months ago and was on maintenance pyridostigmine and steroids. This episode of MG evolved rapidly in our patient. This case is unique because the patient decompensated within 30 minutes of plasma exchange and had to be intubated because of respiratory and hemodynamic compromise. Hypotension secondary to TC led to multiorgan failure and hemodialysis, which ultimately led to death. As per previous literature, there has been at least one other case where an older woman developed TC while undergoing Plasmapheresis for her MC [9]. Although coronary angiography is the gold standard for diagnosing TC, it was not performed due to the clinical instability of the patient.

In terms of treatment, there are no specific therapies against TC. Non-catecholamine inotropes that work by myocardial calcium sensitization like levosimendan can be more appropriate in patients with TC presenting with cardiogenic shock, ionotropic drugs are contraindicated. The management of TC is currently supportive. Intensivists can use an intra-aortic balloon pump or left ventricular assist as bridging therapy during the crisis. To manage MC, plasmapheresis, and intravenous immunoglobulin (IVIG) therapies are among the most common treatment in addition to pyridostigmine and steroids. It is essential to be vigilant about the medication that can worsen myasthenic crisis because a few of them are used in TC treatment. These include beta-adrenergic agonists, calcium channel blockers, procainamide, and quinidine.

## Conclusions

Although myasthenia gravis is a neuromuscular disease, it has cardiac complications. To conclude, we would point out that clinicians are wary of these cardiac complications associated with myasthenia gravis and myasthenia crisis. Close cardiac monitoring with serial electrocardiograms (EKG), cardiac enzymes, and echocardiograms can help diagnose these fatal cardiac complications.
